# Regulation of bone metabolism by megakaryocytes in a paracrine manner

**DOI:** 10.1038/s41598-020-59250-6

**Published:** 2020-02-10

**Authors:** Young-Sun Lee, Mi Kyung Kwak, Sung-Ah Moon, Young Jin Choi, Ji Eun Baek, Suk Young Park, Beom-Jun Kim, Seung Hun Lee, Jung-Min Koh

**Affiliations:** 1 Asan Institute for Life Sciences, 88 Olympic-Ro 43 gil, Songpa-gu, Seoul, 05505 Korea; 2 Division of Endocrinology and Metabolism, Asan Medical Center, University of Ulsan College of Medicine, 88 Olympic-Ro 43 gil, Songpa-gu, Seoul, 05505 Korea; 3 Division of Endocrinology and Metabolism, Department of Internal Medicine, Hallym University Dongtan Sacred Heart Hospital, 7, Keunjaebong-gil, Hwaseong-Si, Gyeonggi-Do 445-907 Korea; 4 Department of Orthopedic Surgery, Asan Medical Center, University of Ulsan College of Medicine, 88 Olympic-Ro 43 gil, Songpa-gu, Seoul, 05505 Korea

**Keywords:** Biochemistry, Biochemistry, Cell biology, Cell biology

## Abstract

Megakaryocytes (MKs) play key roles in regulating bone metabolism. To test the roles of MK-secreted factors, we investigated whether MK and promegakaryocyte (pro-MK) conditioned media (CM) may affect bone formation and resorption. K562 cell lines were differentiated into mature MKs. Mouse bone marrow macrophages were differentiated into mature osteoclasts, and MC3T3-E1 cells were used for osteoblastic experiments. Bone formation was determined by a calvaria bone formation assay *in vivo*. Micro-CT analyses were performed in the femurs of ovariectomized female C57B/L6 and Balb/c nude mice after intravenous injections of MK or pro-MK CM. MK CM significantly reduced *in vitro* bone resorption, largely due to suppressed osteoclastic resorption activity. Compared with pro-MK CM, MK CM suppressed osteoblastic differentiation, but stimulated its proliferation, resulting in stimulation of calvaria bone formation. In ovariectomized mice, treatment with MK CM for 4 weeks significantly increased trabecular bone mass parameters, such as bone volume fraction and trabecular thickness, in nude mice, but not in C57B/L6 mice. In conclusion, MKs may secrete anti-resorptive and anabolic factors that affect bone tissue, providing a novel insight linking MKs and bone cells in a paracrine manner. New therapeutic agents against metabolic bone diseases may be developed from MK-secreted factors.

## Introduction

Bone metabolism is regulated mainly by the action of bone-resorbing osteoclasts and bone-forming osteoblasts. Increased bone resorption and/or decreased bone formation can lead to reduced bone mass and quality, resulting in high fracture risk. Typical conditions that induce this imbalance include estrogen deficiency and immobilization^1,2^. On the contrary, decreased bone resorption and/or increased formation with pharmacological interventions can reverse these imbalances. Synchronously inhibiting bone resorption and stimulating bone formation are regarded as an ideal therapeutic strategy against metabolic bone diseases, such as osteoporosis.

Bone marrow, where most osteoclasts and osteoblasts exist, also contains many types of hematopoietic cells. The coexistence of the cells in the bone marrow allows these cells to influence one another by cell-to-cell contact or in a paracrine manner. Specifically, megakaryocytes (MKs), as one of hematopoietic cell residing in bone marrow, have been intriguing in the field of bone research. MKs, which are polyploid cells derived from MK/erythroid progenitors, generate platelets, which contribute to hemostasis and produce a number of growth factors^3–5^. Interestingly, estrogen deficiency and immobilization reduced the number of MKs^6,7^, and estrogen treatment increased MK number in postmenopausal women^8^. In addition, MK-related disorders are associated with osteosclerosis^9–11^. Thus, MKs may have a critical role in bone metabolism.

Actually, it has been reported that MKs may act on both bone resorption and bone formation. A mouse model with increased MKs showed decreased osteoclast number and bone resorption^12^. *In vitro* experiments have shown that MKs inhibit osteoclast precursors from differentiating into osteoclasts, resulting in the suppression of bone resorption^13–15^. Regarding bone formation, several animal models with increased MK have also shown to exhibit increased bone formation^16^; the increased osteoblast proliferation by MKs mainly mediated it^16–18^. Eventually, the mouse models with increased MK number exhibited the osteosclerotic phenotype^12,16,19–25^. Thus, MK plays an osteoprotective role not only by inhibiting bone resorption, but also by stimulating bone formation, making it an ideal therapeutic target for metabolic bone diseases.

It was reported that MK-derived secreting factors suppress bone resorption, at least in part. *In vitro* experiments showed that MK conditioned media (CM) suppress osteoclastogenesis and/or bone resorption, although the factors have been unidentified until now^13,14^. A mouse model with an increased number of splenic MKs without an alteration of bone marrow MKs showed high bone mass with decreased bone resorption^12^. Secreted factors from MKs rather than MKs themselves may be preferred to develop an anti-osteoporotic agents, because the discovered factors could be easily modified into a pharmacological form. Thus, an anti-resorbing agent can be developed from MK secretions. In contrast, it has been reported that MK stimulates bone formation mainly by direct cell-to-cell contact^16,17^, but it has remained unclear until now whether factors secreted from MK may also stimulate bone formation. Thus, we investigated the role of MK CM in bone resorption and formation in more detail. In addition, to our knowledge, there is no report about an *in vivo* anti-osteoporotic effect of megakaryocyte-secreting factors, thus we also tested it.

## Results

### Suppression of bone resorption by MK CM

**Figure 1:**
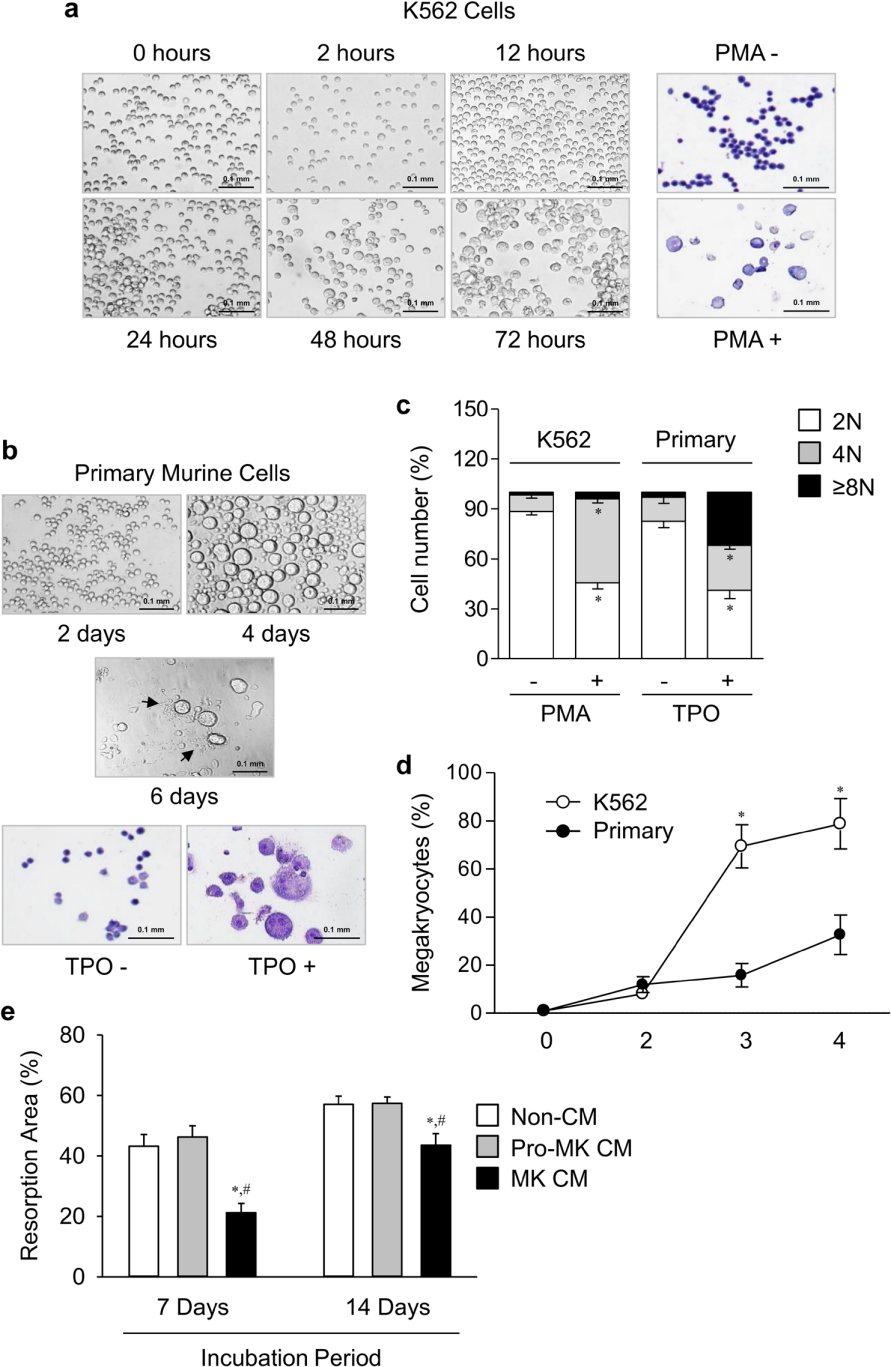
Differentiation of MK cells and suppression of bone resorption by MK CM. (**a**) K562 cells were incubated with phorbol 12-myristate 13-acetate (PMA) for the indicated times. (**b**) Cells from mouse fetal liver were differentiated with thrombopoietin (TPO) for the indicated days. Arrows indicate proplatelet-bearing megakaryocytes (MKs). Cell morphology was observed by microscope, and MK differentiation was detected by Wright-Giemsa staining at 3 and 4 days, respectively. Scale bars, 100 μm. (**c**) DNA polyploid content was analyzed by flow cytometry. K562 and primary cells were treated with PMA or TPO for 3 days or 4 days, respectively. The percentage of cells in each ploidy (2 N, 4 N, and ≥8 N) was shown. (**d**) Differentiation rates of K562 and primary cells. Mature MKs were scored by counting larger than 25 µm in diameter and extensive multinuclearity. (**e**) A resorption pit formation assay of mouse bone marrow macrophages (BMMϕ) cultured with M-CSF and RANKL to form osteoclasts in the presence or absence of 10% (v/v) conditioned media (CM) of MKs and pro-megakaryocytes (pro-MKs) on a dentine disc for the indicated times. MKs were derived from K562 cells. Resorbed areas were quantified as percentages of the total area. Data are presented as mean ± SEM. *P < 0.05 *vs*. non-conditioned media (non-CM, α-MEM media) or primary cells; ^#^*P* < 0.05 *vs*. pro-MK CM. NS, not significant.

Before testing effects of MK CM on bone metabolism, we compared the phenotype and polypoid content of the generated MK from human K562 cell lines and primary murine cells. The K562 and mouse fetal liver cells were differentiated into MK with phorbol 12-myristate 13-acetate (PMA) and thrombopoietin (TPO), respectively. Both cells were successfully differentiated into MKs, based on their cell size and multinuclearity (Fig. 1a,b). The primary cells generated platelets (Fig. 1b), but the MK cells did not (Fig. 1a). A DNA ploidy analysis showed that the primary cells had more polypoid contents than the K562 cells (Fig. 1c). In contrast, differentiation rates markedly higher in K562 cells than primary cells (Fig. 1d). The K562 cells were primarily used in the following experiments, to avoid an excessive sacrifice of mice, to exclude platelet contamination, and to search a human factor in a subsequent study. The primary cells were additionally used to verify several critical findings.

We determined whether MK CM influenced *in vitro* bone resorption (Fig. 1e). The effect was compared to that of undifferentiated cells (pro-MK) CM and non-conditioned media (non-CM), respectively. Consistent with results of a previous report^13^, MK CM significantly suppressed bone resorption, regardless of the incubation duration. Pro-MK CM did not suppress bone resorption, compared with the untreated control.

**Figure 2:**
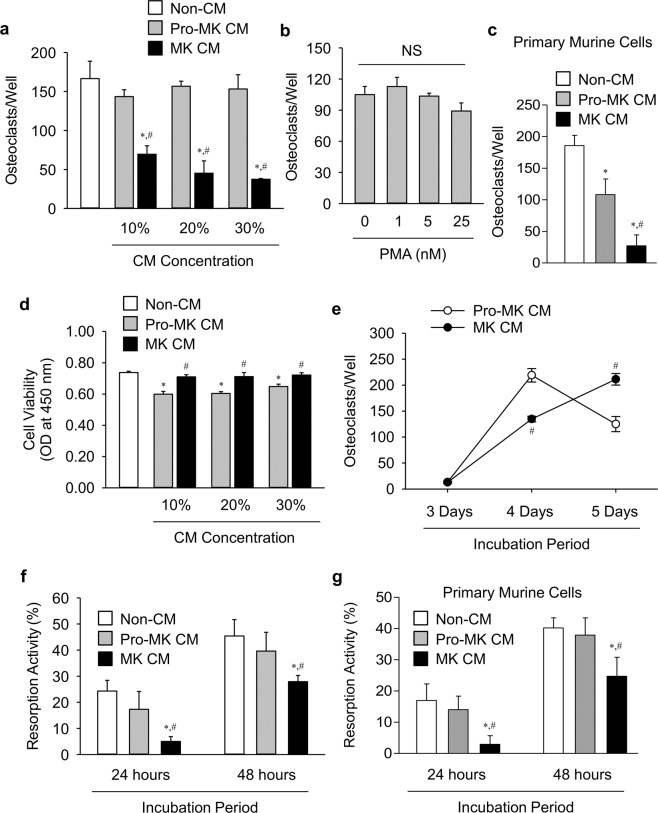
Suppression of osteoclastic resorption activity by MK CM. (**a**) Tartrate-resistant acid phosphatase (TRAP) staining of osteoclasts in the presence or absence of the indicated doses of conditioned medias (CMs) for 4 days. TRAP-positive cells with more than three nuclei were counted. (**b**) TRAP staining of osteoclasts in the presence of the indicated doses of phorbol 12-myristate 13-acetate (PMA) for 4 days. (**c**) TRAP staining of osteoclasts in the presence or absence of 30% (v/v) MK and pro-MK CM fractions derived from murine fetal livers for 4 days. (**d**) Viability of mouse BMMϕ was assessed using a CCK-8 assay in the presence or absence of the indicated doses of CM for 48 hours. (**e**) TRAP staining of osteoclasts in the presence of 10% (v/v) MK and pro-MK CMs for the indicated times. (**f**,**g**) Resorption activity of osteoclasts. After the full differentiation of BMMs into osteoclasts, cells were seeded on a dentine disc with M-CSF and RANKL, and then cultured in the presence of 10% (v/v) MK or pro-MK CM for the indicated times. MK and pro-MK cells were derived from K562 cells (**f**) or from murine fetal livers (**g**). Resorbed areas were quantified as percentages of the total area. MK and pro-MK were derived from K562 cells, unless otherwise specified. Data are presented as mean ± SEM. *P < 0.05 *vs*. non-CM (α-MEM media); ^#^*P* < 0.05 *vs*. pro-MK CM. NS, not significant.

Bone resorption is regulated by osteoclasts, thus we determined their effects on osteoclastogenesis (Fig. 2a). MK CM, but not pro-MK CM, significantly suppressed osteoclastogenesis even at a low concentration. Although we collected the MK CM without PMA, to completely exclude the possibility that remaining PMA contamination may affect this result, we tested whether PMA itself may suppress osteoclastogenesis. Variable concentrations of PMA did not affect osteoclastogenesis (Fig. 2b). Unlike K562 cells, the primary pro-MK CM suppressed osteoclastogenesis compared with non-CM (Fig. 2c). However, primary MK CM more suppressed it than pro-MK CM.

Osteoclastogenesis is influenced by alterations in the pool of osteoclast precursors and/or osteoclastic differentiation. MK CM did not reduce the viability of bone marrow macrophages (BMMϕ), an osteoclast precursor, compared with the effects of non-CM (Fig. 2d), suggesting that MK CM-mediated osteoclastogenesis suppression may be independent of any alteration in the osteoclast precursor pool. Rather, pro-MK CM reduced BMMϕ viability compared with that observed upon treatment with non-CM or MK CM. In other words, MK CM reduced osteoclastogenesis (Fig. 2a) and increased BMMϕ (Fig. 2d) compared with pro-MK CM. Thus, we compared the changes in osteoclastogenesis upon various incubation durations (Fig. 2e). MK CM suppressed osteoclastogenesis at 4 days, but stimulated it at 5 days, compared with pro-MK CM. These results suggest that MK CM may just delay, but not suppress, osteoclastogenesis, compared with pro-MK CM. Thus, it seems unlikely that the difference in final bone resorption between MK and pro-MK CM resulted from any alteration in osteoclastogenesis.

Finally, resorption activity per an individual osteoclast was compared (Fig. 2f). MK CM, but not pro-MK CM, significantly suppressed resorption activity. The same finding was noted with primary murine cells (Fig. 2g). Thus, MK-secreted factors may suppress bone resorption, largely by decreasing osteoclastic resorption activity.

### Stimulation of bone formation by MK CM

**Figure 3:**
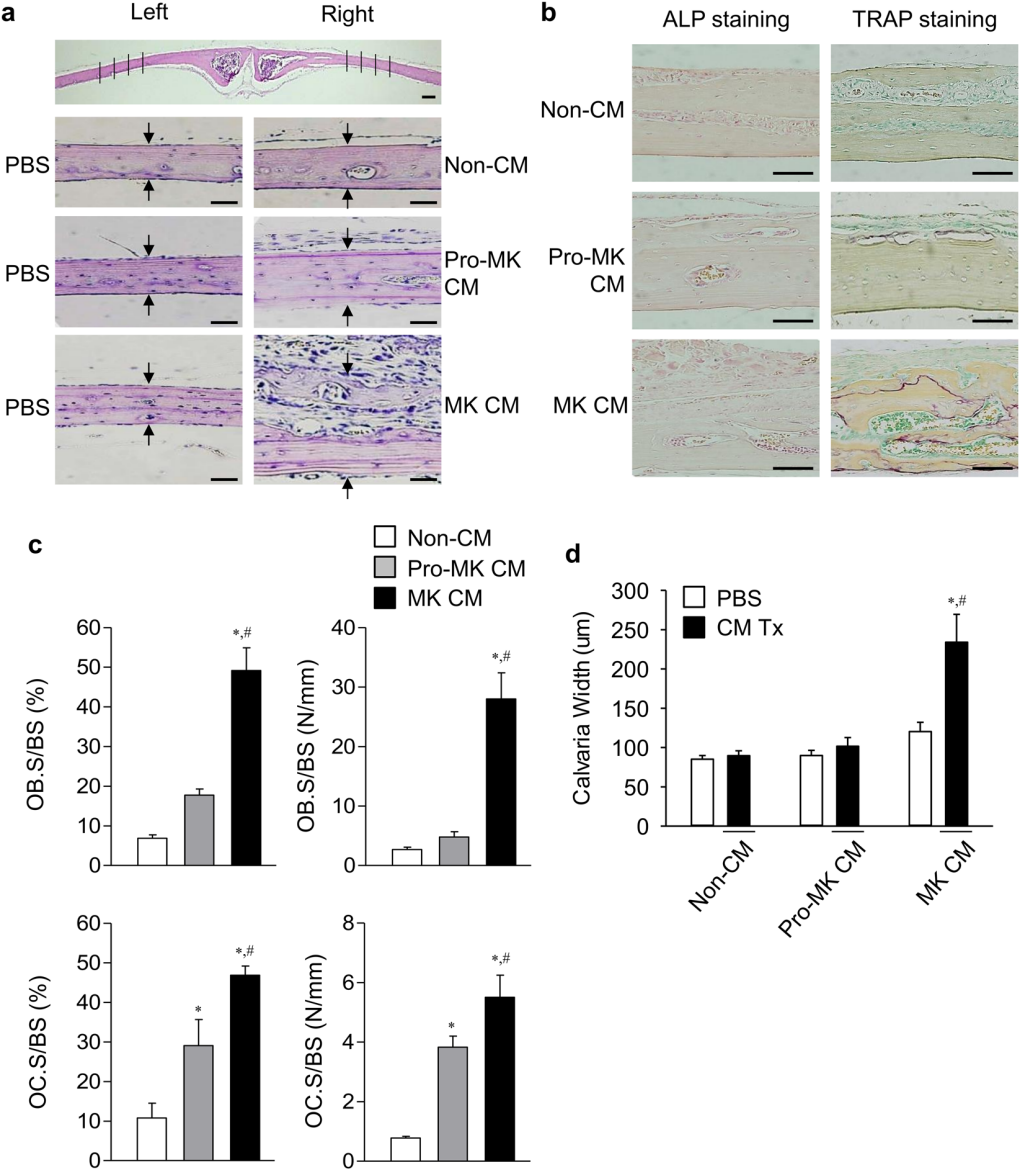
Stimulation of calvaria bone formation by MK CM. 30× Megakaryocyte (MK) and pro-megakaryocyte (pro-MK) conditioned media (CM), and non-CM were injected on the right side of the calvaria in 5-week-old female C57BL/6 mice for 4 weeks. PBS was injected on the left side of the calvaria as a negative control. Hematoxylin and eosin staining (**a**), and alkaline phosphatase immune histochemical (ALP, brown) and tartrate-resistant acid phosphatase (TRAP, purple) stainings (**b**) were performed with the sections of calvaria. (**c**) The osteoblastic surface percentage (OB.S/BS, %) and number per bone surface (OB.S/BS, N/mm), and the osteoclastic surface percentage (OC.S/BS, %) and number per bone surface (OC.S/BS, N/mm), were shown. (**d**) The width of the calvaria was quantitated by the average of 4 spots with the same interval (the indicated lines) of the midline between the sagittal suture and the site of muscle attachment. Arrows indicates osteoid lines as references of calvaria width measurements. Data are presented as mean ± SEM. *P < 0.05 *vs*. non-CM (α-MEM media); ^#^*P* < 0.05 *vs*. pro-MK CM. Scale bar, 50 μm.

To determine their effects on *in vivo* bone formation, the MK and pro-MK CMs were injected onto one side of mouse calvaria bone (Fig. 3). Compared with non-CM and the contralateral side of bone, pro-MK CM did not affect calvaria bone formation, but MK CM increased it (Fig. 3a). Alkaline phosphatase (ALP) immunohistochemical (IHC) staining confirmed that newly formed tissue was bone formed by osteoblasts, showing ALP positivity (Fig. 3b). Tartrate-resistant acid phosphatase (TRAP) staining showed that MK CM increased osteoclastogenesis in calvaria bone (Fig. 3b). This may reflect an increased later osteoclastogenesis with MK CM as shown in Fig. 2e, or secondary phenomenon associated with the elevated osteoblastogenesis. Consistently, the numbers of osteoblasts and osteoclasts were higher in the MK CM-injected sites (Fig. 3c). We measured quantitatively calvaria bone width (Fig. 3d). Compared with the contralateral side, MK CM treatment increased calvaria bone thickness by 1.9-fold.

**Figure 4:**
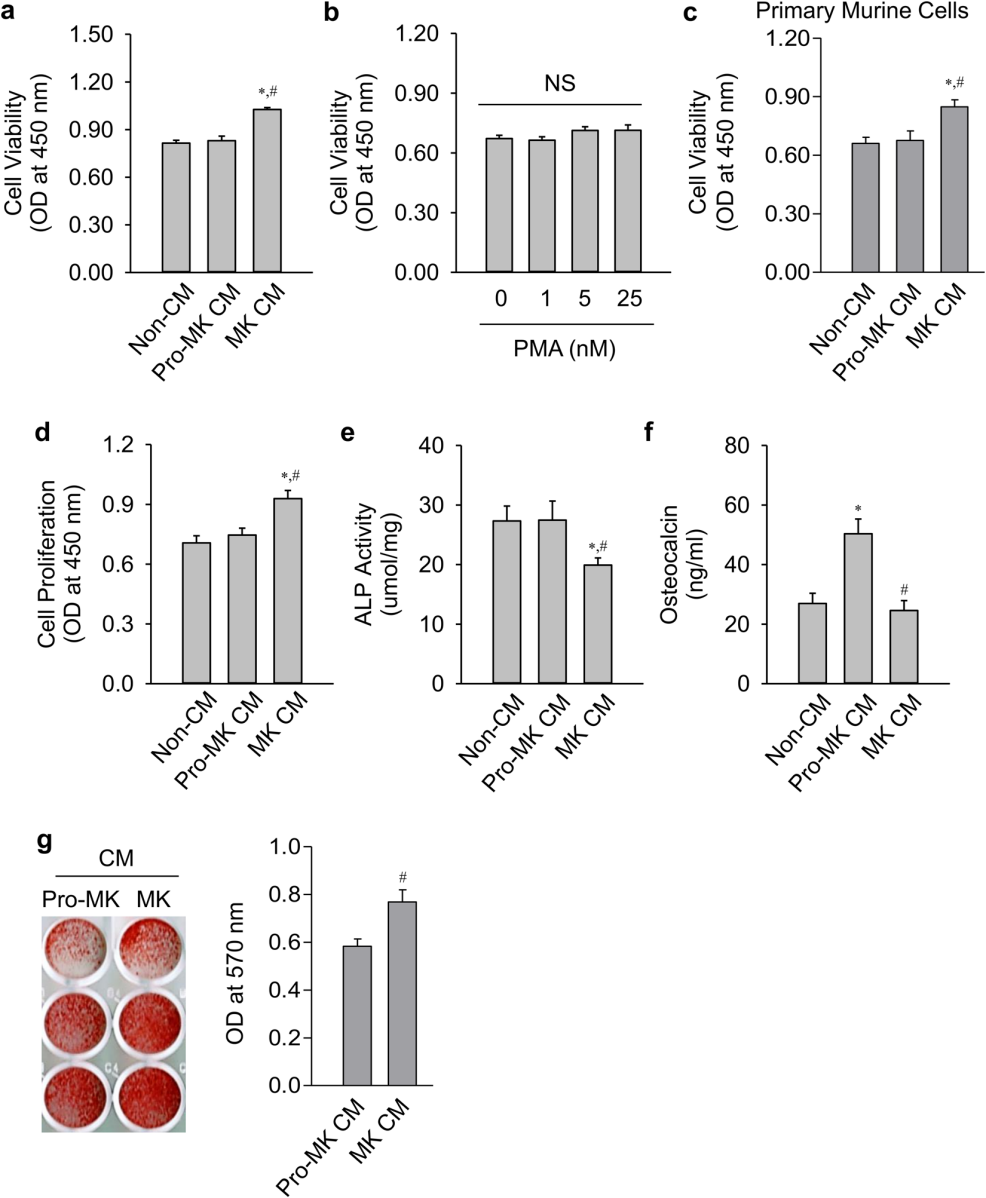
Effects of MK CM on osteoblastic proliferation and differentiation. (**a**) Viability of pre-osteoblast MC3T3-E1 cells was assessed using a CCK-8 assay in the presence or absence of 50% (v/v) conditioned media (CM) from megakaryocytes (MK) or pro-megakaryocytes (pro-MK) for 48 hours. MK and pro-MK cells were derived from K562 cells. (**b**,**c**) Viability of MC3T3-E1 cells was also determined with the indicated doses of phorbol 12-myristate 13-acetate (PMA) (**b**) or 50% (v/v) CMs of enriched MK and pro-MK derived from murine fetal livers (**c**) for 48 hours. (**d**) Proliferation of MC3T3-E1 cells was assessed using a BrdU incorporation assay in the presence or absence of 50% (v/v) MK and pro-MK CMs for 48 hours. (**e**,**f**) Alkaline phosphatase (ALP) activity (**e**) and osteocalcin secretion (**f**) of MC3T3-E1 cells in the presence or absence of 30% (v/v) MK and pro-MK CMs for 7 days. The ALP activity was normalized by total cellular protein amounts. (**g**) Bone nodule formation assay of MC3T3-E1 cells was assessed by Alizarin red S staining, and were quantified by extraction with cetylpyridinium chloride in the presence or absence of 30% (v/v) MK and pro-MK CMs for 14 days. MK and pro-MK were derived from K562 cells, unless otherwise specified. Data are presented as mean ± SEM. *P < 0.05 *vs*. non-CM (α-MEM media); ^#^*P* < 0.05 *vs*. pro-MK CM. NS, not significant.

Increased bone formation may be resulted from increased bone-forming osteoblast number and/or by increased osteoblastic activity. First, osteoblast cell viability was determined after treatment with the CMs (Fig. 4a). Pro-MK CM did not affect viability when compared with that upon non-CM treatment. In contrast, MK CM significantly increased viability compared with both non-CM and pro-MK CM. Variable concentrations of PMA did not affect cell viability, suggesting that MK-secreting factors, but not PMA contamination, stimulated osteoblast viability in this experiment (Fig. 4b). The same result was noted also with primary murine cells (Fig. 4c). MK CM also stimulated osteoblast proliferation, compared with both non-CM and pro-MK CM (Fig. 4d).

Osteoblast differentiation was determined by assaying ALP activity and osteocalcin secretion. MK CM suppressed ALP activity (Fig. 4e), but not osteocalcin secretion (Fig. 4f), compared with non-CM. Pro-MK CM stimulated osteocalcin secretion, but not ALP activity. Compared with pro-MK CM, MK CM suppressed both ALP activity and osteocalcin secretion, suggesting that MK and pro-MK CMs have distinct effects according to assay methods reflecting variable osteoblastic differentiation stages. Regardless, this suggests that any changes in osteoblastic differentiation may not be a crucial cause for increased bone formation by MK CM treatment. Bone formation is resulted from the sum of osteoblast number and its activity. MK CM stimulated *in vitro* bone nodule formation, compared with pro-MK CM (Fig. 4g). Collectively, these data indicate that MK-secreting factors stimulate bone formation mainly by stimulating osteoblast proliferation.

### Therapeutic effects of MK CM in an osteopenic animal model

**Figure 5:**
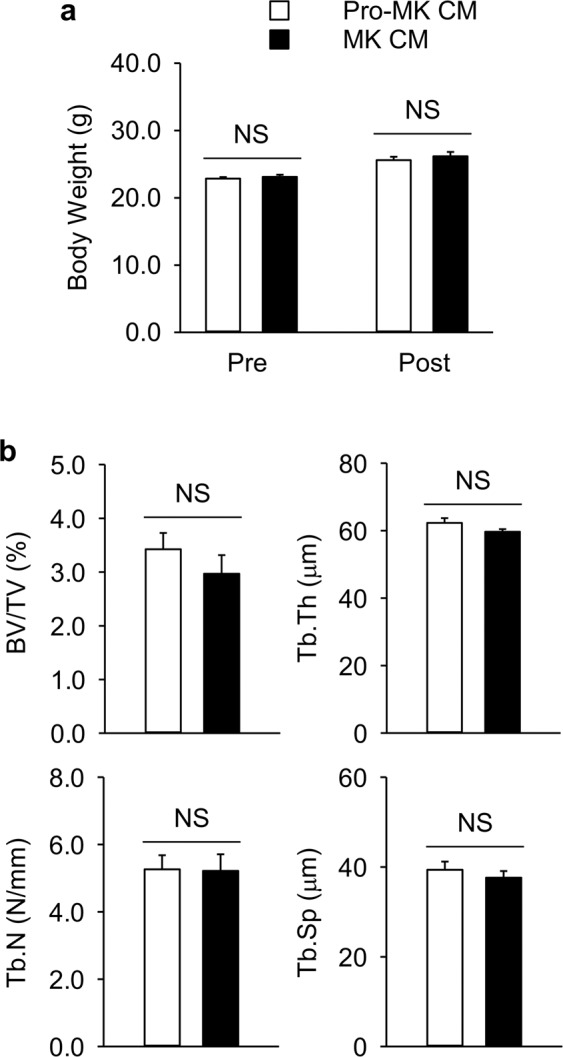
Micro-computed tomography analyses of the femurs in ovariectomized C57B/L6 mice. Female C57B/L6 mice were ovariectomized at 9 weeks of age (n = 8 in each group), and conditioned media (CM) of megakaryocytes (MK) or pro-megakaryocytes (pro-MK) were injected via the tail vein once a day starting at 13 weeks of age for 4 weeks. Mice were then sacrificed for analyses at 17 weeks of age. (**a**) Body weight before (Pre) and after (Post) the injections. (**b**) Trabecular bone parameters of the femurs were also measured. BV/TV, bone volume per tissue volume; Tb.Th, trabecular thickness; Tb.N, trabecular number; Tb.Sp, trabecular separation. Data are presented as mean ± SEM. NS, not significant.

As a pilot study, MK and pro-MK CMs were injected for 4 weeks in OVX C57BL/6 mice. Body weights were similar between the two groups before and after treatments (Fig. 5a). Unexpectedly, MK CM treatment did not improve any bone parameters, compared with pro-MK CM treatment (Fig. 5b). We assumed three possibilities for this result. First, MK CM may not be potent enough to strengthen bones *in vivo*. Second, factors from MK CM may have some pitfalls resulting from their *in vivo* pharmacokinetics or distributions, for example, rapid degradation *in vivo* or insufficient their delivery to bone tissues. Lastly, MK and pro-MK were obtained from human cell lines, and the efficacy of their secreting factors may be eliminated *in vivo* due to the generation of autoantibodies.

**Figure 6:**
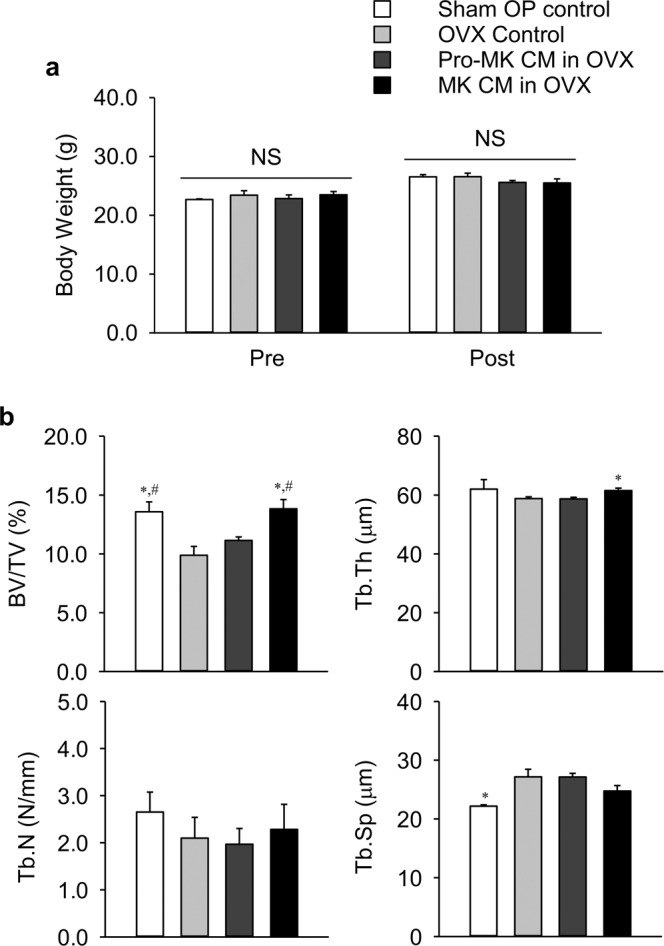
Micro-computed tomography analyses of the femurs in ovariectomized nude mice. Female nude mice were sham-operated or ovariectomized (OVX) at 9 weeks of age (n = 15 in each group), and conditioned media (CM) of megakaryocytes (MK) or pro-megakaryocytes (pro-MK) was injected via the tail vein once a day from 13 weeks of age for 4 weeks. Mice were then sacrificed for analyses at 17 weeks of age. The same volume of saline was injected in the control group. (**a**) Body weight before (Pre) and after (Post) the injections. (**b**) Trabecular bone parameters of the femurs were also measured. BV/TV, bone volume per tissue volume; Tb.Th, trabecular thickness; Tb.N, trabecular number; and Tb.Sp, trabecular separation. Data are presented as mean ± SEM. *P < 0.05 *vs*. untreated control; ^#^*P* < 0.05 *vs*. pro-MK CM. NS, not significant.

To minimize an immune reaction to the CMs, we repeated this *in vivo* experiment in nude mice. The mice were divided into 4 groups: untreated sham-operated mice, untreated ovariectomized (OVX) mice, OVX mice treated with pro-MK CM, and OVX mice treated with MK CM. Body weights were similar among the 4 groups before and after treatments (Fig. 6a). Sham-operated mice had significantly higher bone volume per tissue volume (BV/TV) and lower trabecular spacing (Tb.Sp) than those of untreated OVX mice (Fig. 6b). Pro-MK treatment did not affect any bone parameters, compared with parameters of untreated OVX mice. MK CM treatment increased BV/TV and trabecular thickness (Tb.Th), compared with those of untreated OVX and/or pro-MK CM-treated OVX mice.

## Discussion

We report here that MK CM has distinct roles on bone cells from those of pro-MK CM. MK CM decreased bone resorption by suppressing osteoclastic resorption activity. In addition, MK CM increased bone formation by stimulating osteoblastic proliferation despite suppressing their differentiation. Finally, *in vivo* treatment of MK CM increased bone mass in OVX nude mice. Thus, MK may simultaneously inhibit bone resorption and stimulate bone formation in a paracrine manner.

It has been already reported that MK CM suppressed osteoclastogenesis and bone resorption^13–15^, consistent with our results. In addition, we noted that MK CM treatment increased the number of BMMϕ, an osteoclast precursor, compared with that observed upon pro-MK CM treatment. This suggests that MK CM may delay, but not suppress, osteoclastogenesis. Thus, we cannot be sure that the suppressed bone resorption by MK CM may have resulted from suppressed osteoclastogenesis. Instead, it is reasonable to assume that the suppressed resorption activity of an individual osteoclast may lead to MK CM-suppressed bone resorption, based on the findings shown in Fig. 2f,g. Thus, in order to search novel factors to suppress bone resorption from MK secretions, it may be more suitable to target resorption activity rather than osteoclastogenesis.

We have shown here that MK CM stimulated osteoblastic proliferation and bone formation. This finding contradicts those of the previous reports showing that MK stimulates bone formation by direct cell-to-cell contact, but not in a paracrine manner^16,17^. In the previous reports, co-culture with MK increased osteoblast proliferation, but its CM did not^16,17^. Rather, a higher concentration of MK CM suppressed osteoblast proliferation more^16^. We cannot be sure why an inconsistency was observed between our results and those of previous studies, but one possibility is contamination of TPO in the previous studies, in which cells from mouse liver were differentiated into MK with TPO. In real, it was previously reported that depleting C-Mpl, the receptor for TPO, increases osteoblast proliferation^26^, suggesting that activation of TPO downstream signaling may suppress osteoblast proliferation. In contrast, we differentiated K562 cells into MKs with PMA but without TPO, and further demonstrated that PMA itself did not affect osteoblast viability. Primary murine MK CM was also collected without TPO. Additionally, we cannot exclude the possibility that distinct factors from the different cell types in the previous study and ours may affect osteoblasts differently.

It is well-known that platelet-rich plasma stimulates bone formation and improves the healing process of bone tissue^27,28^. Platelets themselves have no nucleus and thus cannot produce bone-forming factors. Instead, platelets can contain the factors produced by MKs, because they are fragments of mature MK cytoplasm^29,30^. Thus, our finding of stimulated bone formation by MK CM is consistent with the stimulation of bone formation by platelet-rich plasma. Thus, targeting MKs may be useful to search for novel factors that stimulate bone formation.

The most novel finding of our study is an *in vivo* treatment effect of MK CM. We observed that MK CM treatment increased bone mass in nude mice, but not in C57BL/6 mice. The MKs were obtained by differentiation from human K562 cell lines. Thus, it seems likely that an immune reaction against human factors may have led to the lack of therapeutic effect in C57BL/6 mice with normal immunity. In contrast, T cells, which help generate antibodies, were depleted in athymic nude mice. Thus, the therapeutic effect of MKs may be noted in only nude mice with less ability to generate autoantibodies.

However, regardless that MK CM can not only suppress bone resorption, but also stimulate bone formation, the *in vivo* effects of MK CM are not strong enough to be expected. Several reasons may contribute to this phenomenon. First, some immune reactions may still occur in nude mice. Second, MK-secreted factors may be rapidly degraded *in vivo*. For example, the factors may be proteins or peptides which can be easily destroyed by the many proteinases present in circulation. Third, MK may secrete complex factors with catabolic and anabolic actions, thus these combined effects may lead to therapeutic limitations. Last, the nude mice are not a good animal model for estrogen deficiency-induced osteopenia^31^. The T lymphocytes are one of cells mediating osteoclast activation by estrogen deficiency^31^. However, the nude mice cannot generate T lymphocytes. In fact, ovariectomized nude mice did not show a dramatic bone loss compared with sham-operated nude mice in our experiment. Thus, an effect of MK CM on bone resorption may be attenuated in our experiment, and this is a limitation of this study. The treatment with murine MK CM in mice with normal immunity may be more proper approach from this point of view.

Regarding to bone formation, the results of *in vitro* and calvaria bone experiments were consistent. MK CM stimulated osteoblastic proliferation and bone formation *in vitro*, and ALP positivity in calvaria. However, we noted somewhat inconsistent result between the two experiments regarding to bone resorption. MK CM suppressed bone resorption *in vitro*, but increased TRAP positivity in calvaria. However, this is not surprising, given that MK CM stimulated later osteoclastogenesis *in vitro* as shown in Fig. 2e, and that TRAP positivity reflects osteoclast but not bone resorption. In addition, the increased osteoblastogenesis by MK CM may stimulate *in vivo* osteoclastogenesis in a paracrine manner, possibly by affecting RANKL/OPG system of osteoblasts. Anyway, this means that it may be more suitable to target bone formation rather than resorption to search a beneficial factor from MK secretome. Regarding to this issue, lack of a dynamic histomorphometric data is a limitation of this study.

In the present study, human K562 cells were primarily used, as the following reasons. First, it was reported that TPO itself can affect bone cell biology^26^, thus we concerned any confusion to interpret our results. Second, this study is a preceding one to find a human MK-secreting factor. A cell line may be preferred to search a factor than primary cells, to minimize contaminations of other cell types during an omics approach. Third, it is known that platelet itself has an action in bone metabolism^27,28^, thus we wanted to eliminate its effects. Primary cells, but not K562 cells, can generate platelets as shown in Fig. 1a,b. Lastly, we used a cell line to avoid an excessive sacrifice of mice. However, the primary murine cells are more physiologic ones than the K562 cells, and many experiments is based on a xenogenic model to raise an issue about that the results may be influenced by the cross-linking experimental design between human and mouse. Although several critical experiments were verified with primary murine cells, it should be pointed out as a limitation of this study.

Collectively, our study provides a novel insight linking MKs and bone cells, showing that MKs may secrete anti-resorptive and/or anabolic factors. Thus, therapeutic targets against metabolic bone diseases may be discovered from MK-secreted factors.

## Material and Methods

### Animal care

All mice were maintained under specific pathogen-free conditions at the Asan Institute for Life Sciences (Seoul, Korea) and exposed to a 12 hours light-dark cycle. Rodent chow and water were given *ad libitum*. All mice were sacrificed by cardiac puncture under anesthesia with an intraperitoneal injection of 40 mg/kg Zoletil 50 (Virbac, Carros, France) and 5.6 mg/kg Rompun (Bayer Korea, Seoul, Korea)^32^. No specific inclusion or exclusion criteria were used in our animal studies. All methods for animal care and experimental procedures were reviewed and approved by the Institutional Animal Care and Use Committee of the Asan Institute for Life Sciences (No. 2016-12-035). The committee abides by the institute of Laboratory Animal Resources (ILAR) guide. All experiments were done, according with the Korean Ministry of Food and Drug Safety (MFDS) guidelines.

### Megakaryocytes culture and collections of their CM

Megakaryocyte-like cells were generated from the human leukemia cell line K562 at a density of 3 × 10^5^/ml (ATCC, Manassas, VA) by incubation for 3 days with 1 nM PMA (Sigma-Aldrich, St. Louis, MO) in RPMI 1640 medium (Hyclone, Logan, UT) containing 10% fetal bovine serum (FBS; Gibco, Grand Island, NY), 10 U/ml penicillin, and 10 μg/ml streptomycin (Gibco). And then, the CM was collected after further incubation for 24 hours without PMA in serum- and phenol red-free α-MEM. This CM was regarded as mature MK CM. The MK CM from K562 cells were collected, when the cells with ≥ 25 µm of its diameter and 4 N DNA were more than 80% and 50%, respectively. The cells incubated without PMA were regarded as Pro-MK, and their CM was collected similarly to MK CM.

Primary murine MKs were prepared as previously described^33^. In brief, mouse fetal livers of 15.5 gestation days were collected, and single cell suspensions made by forcing cells through sequentially smaller gauge needles. Cells were cultured with DMEM with 10% FBS and murine TPO (50 ng/ml, R&D Systems Inc, Minneapolis, MN**)**. After 3–4 days, the cells were isolated using a one-step albumin gradient by fractions of enriched MKs and depleted MKs. These referred to as the MK and pro-MK fractions, respectively. And then the CM was collected after further incubation for 24 hours without TPO in serum- and phenol red-free α-MEM.

The obtained CM and non-CM was filtered through a 0.45 µm membrane filter. The CMs were stored at −80 °C till use. The CMs were used at 10~50% (v/v) for *in vitro* study. The lyophilized CM was made up at 15× or 30× with DDW for *in vivo* study.

### Assay for cell differentiation

For morphological assessment, cells were stained with Wright-Giemsa staining solutions (Sigma-Aldrich). The cells were explored to show a marked increase in cell size and extensive multinuclearity^34–37^. DNA ploidy analysis was also evaluated by flow cytometry. Cells were collected and fixed with cold 70% ethanol for 1 hour. And then propidium iodide (Sigma-Aldrich) with RNase (Sigma-Aldrich) were added to stain DNA for 30 min, and analyzed by BD FACS Canto II (BD, San Diego, CA).

### Osteoclast differentiation

Bone marrow cells were obtained by flushing the femurs and tibias of 6-week-old ICR mice, and cultured at 37 °C in α-minimum essential medium (α-MEM; Wel Gene, Daegu, Korea) containing 10% FBS, 100 U/ml penicillin, and 100 μg/ml streptomycin in a humidified atmosphere with 5% CO_2_^32^. After 24 hours of culture, non-adherent cells (BMMϕ) were collected, seeded at a density of 4 × 10^4^ cells/well in 96-well culture plates, and were fully differentiated into osteoclasts by culturing with 15 ng/ml macrophage colony-stimulating factor (M-CSF, R&D Systems) and 15 ng/ml soluble receptor activator of nuclear factor-κB (NF-κB) ligand (RANKL, R&D Systems) for 4 days; culture medium was changed every 2–3 days. Adherent cells were fixed and stained using a tartrate-resistant acid phosphatase (TRAP) staining kit (leukocyte acid phosphatase kit; Sigma-Aldrich) according to the manufacturer’s instructions. TRAP-positive multinucleated cells containing three or more nuclei were considered to be osteoclasts, and were counted under a light microscope (Olympus, Tokyo, Japan).

### *In vitro* resorption assay

BMMϕ were seeded on dentine discs (IDS Ltd., Boldon, UK) at a density of 3 × 10^4^ cells/well in 96-well culture plates the presence of 30 ng/ml M-CSF and 30 ng/ml RANKL for 7 and 14 days^38^. The cells on the dentine discs were completely removed by wiping with a cotton swab, and then the dentine slices were stained with hematoxylin (Sigma-Aldrich) for 1 minute. The area of resorbed pits was analyzed using Image-Pro Plus software (MediaCybernetics, Silver Spring, MD).

To evaluate resorption activity of an individual osteoclast, fully differentiated osteoclasts (3 × 10^4^ cells/well in 96-well culture plates) were seeded on dentine discs for 24 and 48 hours, and the resorbed area was measured by the same method described above.

### Cell viability and proliferation assay

BMMϕ (4 × 10^4^ cells/well) or M3CT3-E1 cells (5 × 10^3^ cells/well) were seeded in 96-well culture plates. Cell viability was measured using a commercially available Cell Counting Kit-8 (CCK-8; Dojindo, Kumamoto, Japan) according to the manufacturer’s instructions. Briefly, 10 μl of CCK-8 was added to each well in a 96-well plate for 1 hour, and the absorbance at 450 nm was then read using a microplate reader (SPECTRAmax 340PC; Molecular Devices, Palo Alto, CA) with a reference wavelength of 650 nm^32,38^. Cell proliferation was measured using a 5-bromo-2′-deoxyuridine (BrdU) assay. Cells were incubated with BrdU for 6 hours, and then cell proliferation was assayed using a BrdU labeling and detection kit (Roche, Mannheim, Germany).

### Osteoblast differentiation

Murine pre-osteoblast MC3T3-E1 cells (ATCC) were cultured at 37 °C in α-MEM containing 10% FBS, 100 U/ml penicillin, and 100 μg/ml streptomycin in a humidified atmosphere with 5% CO_2_^32^. The medium was changed every 2–3 days. Upon reaching 80% confluence, cells were sub-cultured with trypsin-EDTA (Gibco). The cells were differentiated into osteoblasts with 50 μg/ml ascorbic acid and 10 mM β-glycerophosphate (Sigma-Aldrich).

ALP activity, osteocalcin secretion, and bone nodule formation were used to measure osteoblastic differentiation. Briefly, MC3T3-E1 cells were seeded at a density of 1 × 10^5^ cells/well in 12-well culture plates, and were differentiated into osteoblasts for 7 days. The cells were washed with PBS and the ALP activity was measured using the p-nitrophenyl phosphate hydrolysis method^39^. The ALP activity in each sample was normalized relative to total cellular protein content, which was determined by the BCA method (Pierce, Rockland, IL). To determine the osteocalcin concentration, the culture medium was collected and osteocalcin was measured using an osteocalcin ELISA kit (BT-470, Alfa Aesar, Ward Hill, MA).

Bone nodule formation was assessed at 14 days by Alizarin red S (ARS) staining. For ARS staining, cells were fixed in ice-cold 70% ethanol and stained for 15 min with 40 mM ARS (Sigma-Aldrich) at pH 4.2 and room temperature. The stained cells were rinsed three times with distilled water, and the bound ARS was eluted with 10% cetylpyridinium chloride at pH 7.0 (Sigma-Aldrich). Extracted ARS samples were quantified by measuring absorbance at 570 nm.

### *In vivo* calvaria bone formation

C57BL/6 mice at 5 weeks of age were used. A total of 50 µl of 30× MK and pro-MK CMs were injected subcutaneously over the right parietal bone using a 31-gauge needle once daily for 4 weeks. Mice were sacrificed after one week, then the calvaria bones were fixed in 4% paraformaldehyde for 24 hours and decalcified in 0.5 M EDTA in PBS for 2 to 4 weeks^40^. Decalcified specimens were embedded in paraffin, and then sectioned coronally at 6 μm. After deparaffinization, the sections were rehydrated, and hematoxylin and eosin (H&E, Sigma-Aldrich), ALP (Sigma-Aldrich) IHC, or TRAP (Sigma-Aldrich) staining was performed following the manufacturer’s instructions. The calvaria bone widths were measured at 4 adjacent spots with the same interval of the midline between the sagittal suture and the site of muscle attachment using Image-Pro Plus software (Media Cybernetics), and its mean values were presented.

### Systemic treatment with MK CM in ovariectomized mice

Female C57BL/6 J and Balb/c nude mice were bilaterally OVX at 9 weeks of age, and 100 µl of 15× MK or Pro-MK CMs was injected via the tail vein daily from 13 weeks of age for 4 weeks^32^. Mice were sacrificed at 17 weeks of age, and the ovariectomy success was confirmed by observing ovary absence and uterus atrophy. The same volume of PBS (100 µl) was injected in the OVX and sham-operated mice as control groups. All OVX groups were weight-matched at the initiation of injection. The researcher conducting the injections was not blinded to the experimental groups, but the researcher assessing bone parameters was blinded to the groups.

### Micro-computed tomography (micro-CT) of femurs

For three-dimensional morphometric analysis, femurs were scanned using the Skyscan 1172 system (Skyscan, Antwerp, Belgium, Germany) at 50 kV/200 μA with 6.48 μm pixel size and 0.5-Al filters^32^. Reconstructions were performed with NRecon (Skyscan). For analysis of trabecular bone, regions of interest (ROI) of cancellous bone were created within the endosteal envelope on the two-dimensional slices. The ROI extended 3 mm from the growth plate of each femur to the proximal metaphysis, and three-dimensional algorithms were used to determine the relevant parameters. All morphometric parameters were determined using CTan (Skyscan). The coefficient of variation (CV) of BV/TV was 4.9%.

### Statistical analyses

All *in vitro* and *in vivo* data are expressed as mean ± standard error of at least three independent experiments conducted with triplicate measurements unless otherwise specified. The significant differences between two groups were tested using the Mann–Whitney U-test, whereas differences between three or more groups were tested using the Kruskal–Wallis test followed by Bonferroni correction. All statistical analyses were performed using SPSS statistical software (SPSS Inc., Chicago, IL), and *p* values < 0.05 were considered statistically significant.
